# Machine learning quantifies immuno-virological interactions: a TRIPOD+AI compliant prediction model for HIV-1 salvage therapy outcomes

**DOI:** 10.3389/fimmu.2026.1846559

**Published:** 2026-08-05

**Authors:** Defu Yuan, Yangyang Liu, Shanshan Liu, Nana Peng, Xiaoyue Zhu, Qingjin Qian, Bei Wang, Yueqi Yin

**Affiliations:** 1 Department of Epidemiology and Health Statistics, Key Laboratory of Environmental Medicine Engineering of Ministry of Education, School of Public Health, Southeast University, Nanjing, China; 2 Department of Public Health, Zaozhuang Mental Health Center, Zaozhuang, China; 3 Medical Data Analytics Centre, Department of Medicine and Therapeutics, The Chinese University of Hong Kong, Hong Kong, Hong Kong SAR, China; 4 Department of Otolaryngology Head and Neck Surgery, Shanghai Sixth People’s Hospital Affiliated to Shanghai Jiao Tong University School of Medicine, Shanghai, China; 5 Yunnan AlDS Care Center, Yunnan Mental Health Center, Yunnann Infectious Disease Hospital, Kunming, China; 6 Department of Data Center, Yinzhou District Center for Disease Control and Prevention, Ningbo, China; 7 School of Public Health, Ningbo University, Ningbo, China

**Keywords:** genotypic susceptibility score, HIV-1, machine learning, precision medicine, salvage therapy, SHAP interaction analysis, TRIPOD+AI approach

## Abstract

**Background:**

The management of multidrug-resistant HIV-1 in patients experiencing virologic failure remains a critical clinical challenge. Traditional linear scoring systems often fail to adequately capture the complex evolutionary dynamics between the virus, host immunity, and antiretroviral regimens. This study aims to construct and validate a prediction model in line with the TRIPOD+AI statement to quantify the multidimensional, nonlinear interactions among “virus-host-drug” and to guide individualized clinical salvage therapy.

**Methods:**

This retrospective cohort study integrated de-identified data from 18 clinical trials in the Stanford HIV Drug Resistance Database (n = 6,844). Seven machine learning algorithms were compared with traditional models. Model evaluation metrics included area under the receiver operating characteristic curve (AUC), Brier score, and calibration curves. TreeSHAP quantified feature contributions and interactions. Ablation studies (DeLong’s test) evaluated the incremental predictive value of integrating virological, immunological, and treatment history domains. The algorithmic fairness of the model across populations with different immune statuses and viral loads was evaluated through subgroup analysis, and the Effective Sample Size (ESS) was introduced to assess individual prediction uncertainty.

**Results:**

The XGBoost model best predicted 24-week virologic suppression (AUC: 0.887), significantly outperforming the baseline model (AUC: 0.816) with excellent calibration (Brier score: 0.096). Ablation studies confirmed that the integrated model significantly outperformed partial models restricted to single feature domains (all *P* < 0.05). SHAP interaction analysis revealed a significant modification effect of baseline CD4^+^ T cell count on the predictive weight of viral load; meanwhile, a temporal decay in drug resistance test results was observed, significantly diminishing the negative predictive weight of a heavy treatment history. Reclassification analysis showed that the XGBoost model corrected 61.90% of actual failures misclassified by the baseline model, demonstrating a significant net clinical benefit (Net Reclassification Improvement: 0.490). Clinical fairness checks confirmed that the model performed stably in subgroups with severe immune compromise and high viral loads, without showing systematic bias.

**Conclusion:**

The developed XGBoost model overcomes the limitations of traditional linear scoring and achieves precise prediction of HIV salvage therapy outcomes by quantifying immune modulatory effects and therapeutic exhaustion markers. This model acts as a clinical safeguard to identify ineffective treatments while maintaining algorithmic fairness across patient severities. The developed web-based calculator and risk stratification system help clinicians optimize resource allocation and advance novel drug use in complex resistance scenarios, promoting evidence-based HIV precision medicine practices.

## Introduction

Over the past forty years, since the advent of antiretroviral therapy (ART), Human Immunodeficiency Virus (HIV) infection has gradually transformed from a fatal disease to a manageable chronic condition. Joint United Nations Programme on HIV/AIDS (UNAIDS) reports indicate that as of 2024, the global number of people living with HIV (PLWH) has reached 40.8 million, with approximately 36 million PLWH receiving ART (1). Admittedly, ART regimens with higher genetic barriers have successfully decreased overall resistance to older drug classes (2). However, viral evolutionary pressure dictates that the accumulation of resistant strains does not cease but rather shifts toward newer drugs. Recent studies confirm that resistance to second-generation integrase strand transfer inhibitors (INSTIs) is increasing significantly among virologic-failure PLWH (3), gradually weakening the overall ART defense line. Among PLWH receiving ART, approximately 6%, representing over 2 million PLWH, have failed to achieve virologic suppression (1). World Health Organization (WHO) reports similarly show that the potential resistance rate to core drugs such as dolutegravir (DTG) in first-line treatment failure populations has shown an upward trend in some countries, posing a direct threat to the realization of the goal to “end the AIDS epidemic by 2030.” (4, 5) Recent studies using large-scale cohort data have confirmed that although modern antiretrovirals (ARVs) reduce the overall resistance rate through high genetic barriers, the detection rate of the key mutation R263K against INSTIs has shown a significant upward trend in specific treatment-failure populations, being approximately three times higher in 2024 than in 2018 (2). Deep sequencing technology has also revealed an underestimated burden of INSTI resistance mutations among PLWH (6), suggesting that HIV is adapting to the selection pressure of modern ARVs. For PLWH experiencing treatment failure, clinical management goals have been upgraded to achieving complete virologic suppression (viral load < 50 copies/mL), which is not only a physiological requirement to block chronic inflammation and limit the size of the viral reservoir (7) but also the cornerstone of the Undetectable = Untransmittable (U=U) public health concept (8, 9). However, facing the complex mutation profiles of treatment-failure PLWH, how to piece together a salvage therapy regimen from the remaining drugs that can achieve this standard of perfect suppression has become an insurmountable obstacle for traditional empirical medicine (10).

Genotypic drug resistance testing is currently the gold standard method for guiding salvage therapy. Clinically, the effectiveness of salvage regimens is usually evaluated by calculating the Genotypic Susceptibility Score (GSS). This score is primarily based on the HIVdb algorithm of the Stanford University HIV Drug Resistance Database, which quantifies the antiviral activity of drugs against HIV in PLWH (11). Although GSS is the cornerstone of clinical selection of salvage therapy regimens, its core defect lies in its reliance on the linear additivity assumption, which isolates the resistance contributions of each mutation and simply adds them up. Biologically, this method ignores the widespread phenomenon of epistasis in viral evolution, that is, the existence of complex non-linear interactions between mutations. For example, although the widespread M184V mutation confers high-level resistance of HIV to lamivudine (3TC), basic research has shown that it can confer unexpected clinical benefits in specific mutation combinations by improving the replication fidelity of reverse transcriptase and reducing viral fitness (12). Furthermore, as an independent virological indicator, GSS does not account for the non-linear modification of host immune status on drug efficacy, potentially leading to misleading guidance when relying solely on GSS for salvage therapy in PLWH with severely compromised immunity.

In recent years, studies have demonstrated the substantial potential of machine learning (ML) for HIV research. Altmann et al. utilized the European EuResist dataset to show that classifier fusion methods integrating viral genotype and clinical data could effectively predict virological response to ART (13). Revell et al. demonstrated that computational models based solely on clinical history could predict treatment outcomes in resource-limited settings without genotyping (14), while a recent study developed robust models for viral suppression in a West African cohort, highlighting the importance of addressing class imbalance (15).

However, these works leave critical gaps: models that omit genotypic data fail to capture complex viral escape mechanisms, thereby severing the biological interaction between the virus and the drugs. Furthermore, while techniques like oversampling of the minority class are used to handle class imbalance, they can generate clinically unrealistic synthetic profiles when applied to sparse, discrete drug regimen features. Beyond these methodological limitations, previous studies have pointed out that algorithms trained solely on historical medical data may inadvertently solidify racial and health inequalities, leading to the systematic underestimation of risks in marginalized groups (16). Reviews of medical AI literature have repeatedly demonstrated a prevalent lack of strict reporting standards and widespread methodological flaws, which severely compromise model reproducibility and clinical safety (17, 18). Concurrently, while a substantial body of work on explainable AI (XAI) has emerged in medicine to address the “black box” problem (19), the routine integration of these interpretable methods into HIV therapy modeling remains scarce.

Consequently, a critical research gap persists in the current landscape: a lack of high-precision prediction models for HIV salvage therapy that simultaneously capture complex “virus-host-drug” interactions, handle class imbalance without artificial data synthesis, provide patient-level interpretability, and adhere to modern, standardized reporting guidelines. To address these critical deficiencies and bridge this gap, the Transparent Reporting of a multivariable prediction model for Individual Prognosis or Diagnosis (TRIPOD+AI) statement was published in 2024 to update the original 2015 guidelines, explicitly accommodating the unique challenges posed by machine learning methods (20). This framework establishes new, stringent standards anchored in four key domains: transparency, calibration, algorithmic fairness, and standardized reporting. In clinical practice, being TRIPOD+AI compliant requires researchers to make the model’s inner workings interpretable, rigorously assess calibration to ensure predicted probabilities reflect true clinical risk, and proactively evaluate fairness to ensure the algorithm performs consistently across diverse and vulnerable patient subgroups. Therefore, developing a new generation of predictive models that strictly adhere to these expanded standards is the necessary path to safely and ethically integrating AI technology into clinical practice. To overcome the limitations of linear additivity in traditional GSS and improve the credibility of AI models in clinical practice, this study used standardized, de-identified clinical trial data from the Stanford HIV Drug Resistance Database to construct and validate a scalable XGBoost prediction framework that strictly adheres to the TRIPOD+AI statement (20). This framework captures the complex, non-linear synergistic effects among viral genotypic resistance, host immune status, and viral load through ensemble learning algorithms, and integrates SHapley Additive exPlanations (SHAP) interaction analysis (21) to achieve a quantitative elucidation of the “virus-host-drug” non-linear interaction in HIV salvage therapy. Specifically, this study developed a high-precision prediction model for virologic suppression after salvage therapy in treatment-failure PLWH and rigorously assessed its predictive reliability using the Brier score and calibration slope. Meanwhile, this study also evaluated the model’s algorithmic fairness across severe groups, such as the immunocompromised, through clinical subgroup analysis to ensure its applicability in complex cases. In addition, by combining the optimal model with effective sample size (ESS) information, this study developed a salvage therapy success rate prediction platform to provide reference information for selecting salvage therapy regimens.

## Materials and methods

### Data sources and inclusion/exclusion criteria

This study was a retrospective cohort study, reported strictly in accordance with the TRIPOD+AI statement (20); the TRIPOD+AI checklist is available in **Supplementary File 2**. This study integrated individual patient data from 18 clinical trials in the Stanford University HIV Drug Resistance Database, using the treatment-change episodes (TCE) module. The sample size was determined by all available records in the database that met the inclusion/exclusion criteria. Inclusion criteria included: (1) HIV-1 infected individuals who had previously experienced antiretroviral treatment failure; (2) genotypic drug resistance testing was performed before switching ART regimens. Exclusion criteria included: (1) the interval between genotypic drug resistance testing and regimen switch exceeded 2 years (104 weeks); (2) missing baseline HIV-1 RNA viral load or CD4^+^ T cell count at the time of regimen switch; (3) missing viral load test results at 24 weeks (± 4 weeks) after switching the regimen.

### Ethical review and informed consent

The data for this study were de-identified data from the Stanford University HIV Drug Resistance Database. The data were anonymous and publicly available, and this study involved no direct contact with relevant human subjects. According to the institutional guidelines of Southeast University and the Declaration of Helsinki, this study was considered exempt from ethical review and informed consent.

### Genotypic score calculation and study feature construction

This study extracted sequences from each genotypic drug resistance test in the clinical trials and used the latest version of the Stanford HIVdb algorithm (version 10.0-1) to determine the degree of drug resistance. Values were assigned based on susceptibility to the drugs in the switched regimen (Susceptible = 1.0, Potential Low-Level Resistance = 0.75, Low-Level Resistance = 0.5, Intermediate Resistance = 0.25, High-Level Resistance = 0.0) to calculate the GSS, which was used to quantify the regimen’s overall antiviral activity. Additionally, using the longitudinal treatment trajectories in the raw dataset, this study calculated the “Current Switch Count” and “Total Switches” for each patient as indicators characterizing prior treatment complexity and potential adherence. Since the matched genotypic drug resistance test result for each regimen switch record was the most recent one within 2 years, to eliminate potential bias caused by excessive time intervals, this study also constructed a “Genotype Time Quality” variable, categorized into < 6 months, 6–12 months, and 1–2 years, to adjust for the impact of data timeliness. Other features included in the analysis were baseline viral load, baseline CD4^+^ T cell count, number of prior treatment lines, time from genotypic drug resistance testing to the current switch, and specific drugs in the ART regimen. For PLWH who underwent multiple regimen switches, the viral load and CD4 test results corresponding to each regimen switch were also referred to as the baseline viral load and baseline CD4, respectively.

### Outcome definition and sensitivity analysis

The primary outcome of this study was virologic suppression at 24 weeks after treatment, defined as plasma HIV-1 viral load (VL) < 50 copies/mL. To evaluate the robustness of the model across different clinical scenarios, this study established two secondary outcomes for sensitivity analysis: VL < 200 copies/mL and VL < 1000 copies/mL, to compare the predictive efficacy of the models under different thresholds for virologic suppression.

### Prediction model construction and interpretation

Prior to model construction, a Spearman correlation matrix was used to assess correlations among variables, and the Variance Inflation Factor (VIF) was used to assess multicollinearity; any variable with a VIF exceeding 10 was excluded from subsequent analysis. Subsequent feature selection was inherently driven by the algorithms’ information gain criteria during training. This study constructed six models: Logistic Regression, Random Forest, Support Vector Machine, LightGBM, Neural Network, and XGBoost. Additionally, an ensemble model was constructed using a “Soft Voting” strategy, which calculated the arithmetic mean of the predicted probabilities from the best-performing independent models as the final output, to explore the impact of multi-model fusion on predictive performance. The hyperparameters and configuration details of all models are presented in **Supplementary Table 1**.

The dataset was randomly partitioned at an 8:2 ratio based on patient ID, ensuring that all regimen-switch records for the same patient were assigned to the same dataset. To address the imbalance in the primary outcome, characterized by an overall success rate of 19.83%, this study implemented an algorithm-level class weighting strategy. To strictly prevent data leakage, a majority-to-minority weight ratio of 3.91 was calculated solely within the training set by dividing the number of treatment-failure PLWH by the number of virologic suppression PLWH. For a fair methodological benchmark, this compensation factor was applied across all algorithms during training by adjusting specific weighting parameters, aiming to prevent majority-class bias and maintain balanced sensitivity and specificity. Subsequently, 5-Fold Cross-Validation combined with grid search was employed for hyperparameter optimization. The optimal model was selected based on comprehensive performance on the independent test set. Model evaluation metrics included area under the receiver operating characteristic curve (AUC), accuracy, sensitivity, specificity, and the Brier score, which reflects the accuracy of predicted probabilities.

To quantify the net clinical benefit of the optimal model compared to the traditional baseline model, this study calculated the Net Reclassification Improvement (NRI) and the Integrated Discrimination Improvement (IDI). The baseline model was defined as a univariate logistic regression model based solely on the GSS score. Using the optimal classification threshold based on the Youden index, the ability of the optimal model to correct misclassification in both actual treatment success and actual treatment failure populations was evaluated. To verify the internal robustness of the optimal model and to mitigate the risk of overfitting, a balanced 5-fold cross-validation was performed on the training set. The dataset was randomly divided into 5 equal-sized subsets; 4 were used for training in turn, and the remaining one for validation, with AUC fluctuations recorded for each fold.

To assess whether the optimal model exhibited algorithmic bias, a clinical fairness check was performed. PLWH in the test set were divided into different subgroups based on baseline CD4^+^ T cell count (< 200 cells/µL vs. ≥ 200 cells/µL) and baseline viral load (< median vs. ≥ median). The AUC, 95% CI, and Brier score were calculated for each subgroup and visualized using forest plots.

To break the “black box” effect of machine learning models, this study employed the SHAP method to interpret the optimal model. While quantifying feature importance, SHAP interaction plots were further used to explore non-linear synergistic effects between features. To ensure the robustness of interpretations, TreeSHAP was employed to estimate feature attribution based on the expectations over the training data distribution implicit in the tree structure. Furthermore, this study quantitatively assessed the stability of feature attributions by performing a 5-fold cross-validation procedure. Meanwhile, this study evaluated the model’s clinical applicability using calibration curves and Decision Curve Analysis (DCA) (22). To further quantify the model’s calibration performance, the Calibration Intercept and Calibration Slope were calculated to assess the consistency between predicted and observed probabilities, as well as the presence of overfitting. Furthermore, this study introduced ESS as a confidence metric reflecting the amount of effective historical data supporting a specific patient’s prediction, aiming to quantify individual prediction uncertainty. Based on the constructed XGBoost model, the Parametric Bootstrap method was adopted. By resampling to generate 500 simulated datasets and retraining the model, the variance of the predicted probability and ESS for each patient was calculated.

An ablation study was conducted to test whether the optimal model outperforms traditional models based on isolated clinical feature dimensions, using three independent XGBoost submodels trained and evaluated on a single clinical feature domain: (1) the virological and regimen domain, encompassing baseline viral load, GSS score, genotype time quality, and the use of specific ARVs; (2) the immunological domain, utilizing solely the baseline CD4^+^ T cell count; and (3) the treatment history domain, comprising the number of prior treatment lines, total regimen switches, and cross-class switch patterns. Finally, DeLong’s test was used to compare the predictive performance of each submodel with that of the integrated XGBoost model.

### Statistical analysis

Statistical analysis was performed using R software (version 4.5.2). Based on the primary outcome (whether VL < 50 copies/mL was achieved 24 weeks after the regimen switch), the Wilcoxon rank-sum test and Chi-square test were used to compare continuous and categorical variables, respectively. Since some patients switched treatment regimens more than once during the clinical trial period, a Generalized Linear Mixed Model (GLMM) including patient ID as a random intercept was constructed to identify independent predictors of treatment success and adjust for individual patient effects. To prevent the omission of critical confounders and suppressor variables, candidate predictors were included in the multivariable GLMM based on priori clinical relevance and algorithmic feature importance, deliberately avoiding univariate significance pre-screening, as recommended by modern biostatistical consensus (23, 24). To ensure the robustness of the parameter estimates for the prediction model, the Events Per Variable (EPV) ratio was calculated. The final analysis dataset included 1357 events for the primary outcome and approximately 20 candidate predictors. The calculated EPV exceeded 60, which was higher than the statistically recommended minimum standard (EPV > 10). A two-sided P-value ≤ 0.05 was considered statistically significant.

## Results

### Data cleaning

**Figure 1:**
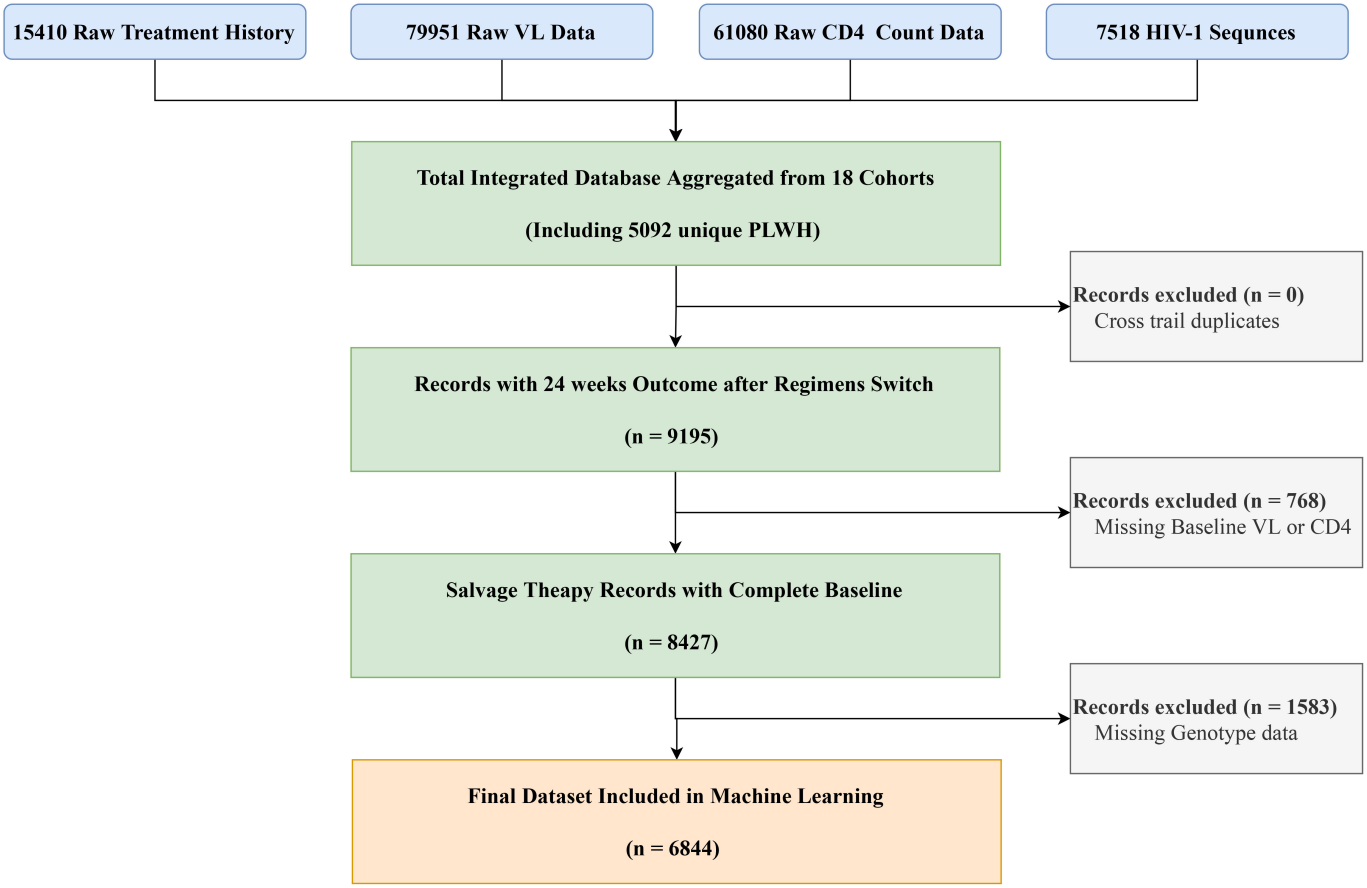
Flowchart of study population selection and exclusion. The diagram illustrates the step-by-step filtering process applied to the dataset from 18 clinical trials, detailing exclusions at each stage to yield the final analysis cohort (n=6,844).

**Figure 1** illustrates the data cleaning process. A total of 79,951 viral load measurements, 61,080 CD4^+^ T cell counts, 7,518 genotypic drug resistance test sequences, and 15,410 ART regimen switch records were extracted from 18 clinical trials. After data cleaning, 9,195 salvage therapy records possessed viral load measurements 24 weeks post-regimen switch. Records were then excluded for two reasons: 1,583 lacked genotypic drug resistance test sequences within 2 years prior to the regimen switch, and 768 lacked baseline viral load or CD4 data. Finally, 6,844 salvage-therapy records were included in the analysis.

### Study population and baseline characteristics

Defining virologic suppression as VL < 50 copies/mL, the treatment success rate was 19.83% (1357/6844). **Table 1** presents the baseline characteristics of the treatment success and failure groups. Significant differences were observed between the two groups regarding VL, CD4 count, number of prior regimens at the time of switching, total number of regimen switches, GSS, and the genotype time quality (P ≤ 0.05). The treatment success group exhibited a lower median VL (4.30 vs. 4.40 log10 copies/mL), a higher median CD4 count (301 vs. 217 cells/µL), fewer median prior regimens (2.00 vs. 3.00), fewer average total regimen switches (2.77 vs. 4.02), a higher proportion of new regimens with a GSS ≥ 3 (81.1% vs. 57.8%), and a higher proportion of genotypic drug resistance test results obtained within 6 months (85.56% vs. 81.70%).

**Table 1:** Characteristics of the PLWH stratified by treatment outcome.

Variants	Viral suppression (<50 copies/mL)		P
	No	Yes	
N	5487	1357	
Baseline VL (median [IQR])	4.40 [3.20, 5.20]	4.30 [3.20, 5.00]	<0.001
Baseline CD4 (median [IQR])	217.00 [105.00, 363.00]	301.00 [151.00, 448.00]	<0.001
Treatment Line (median [IQR])	3.00 [2.00, 4.00]	2.00 [2.00, 3.00]	<0.001
Total Switches (mean ± SD)	4.02 ± 2.71)	2.77 ± 2.03	<0.001
GSS (%)			<0.001
< 1	910 (16.6)	92 (6.8)	
1 - 1.99	518 (9.4)	33 (2.4)	
2 - 2.99	889 (16.2)	132 (9.7)	
≥ 3	3170 (57.8)	1100 (81.1)	
Weeks Since Genotype (median [IQR])	1.00 [0.00, 16.00]	0.00 [0.00, 10.00]	<0.001
Genotype Time Quality (%)			0.001^*^
High Quality (< 6 month)	4483 (81.7)	1161 (85.6)	
Medium Quality (< 1 year)	445 (8.1)	103 (7.6)	
Low Quality (1-2 year)	559 (10.2)	93 (6.9)	

*P ≤ 0.05.

PLWH, People Living with HIV; VL, Viral Load; CD4, CD4+ T cell count; GSS, Genotypic Susceptibility Score; IQR, Interquartile Range.

### Factors influencing salvage therapy efficacy

To maximize information retention, GSS scores and the genotype time quality were included as continuous variables in the GLMM. Correlation analysis indicated no significant collinearity among the variables (Spearman correlation coefficient matrix is shown in **Supplementary Figure 1**). **Table 2** presents the results of the multivariate GLMM analysis. After adjusting for individual patient effects, GSS scores and CD4 counts were identified as protective factors. For every 1-point increase in the GSS of the new regimen, the likelihood of treatment success increased by 1.559 times (OR = 1.559, 95% CI: 1.419-1.712); for every 1 cell/µL increase in baseline CD4^+^ T cell count, the likelihood of treatment success increased by 1.002 times (OR = 1.002, 95% CI: 1.001-1.002). Conversely, VL, the total number of regimen switches, and the genotype time quality were identified as risk factors. For every 1 log10 copies/mL increase in baseline VL, the treatment success rate decreased by 16.4% (OR = 0.836, 95% CI: 0.769-0.910); for every additional regimen switch, the treatment success rate decreased by 24.2% (OR = 0.758, 95% CI: 0.708-0.811); and for every 1-week increase in the interval between drug resistance testing and regimen switching, the treatment success rate decreased by 0.7% (OR = 0.993, 95% CI: 0.988-0.998).

**Table 2:** Generalized linear mixed model analysis for predictors of viral suppression.

Variants	cOR (95% CI)	P value	aOR (95% CI)	P value
GSS Score	1.633 (1.494-1.786)	< 0.001	1.559 (1.419-1.712)	<0.001
Baseline VL	0.947 (0.886-1.013)	0.115	0.836 (0.769-0.910)	<0.001
Baseline CD4	1.001 (1.001-1.002)	< 0.001	1.002 (1.001-1.002)	<0.001
Treatment Line	0.777 (0.729-0.827)	< 0.001	0.980 (0.896-1.072)	0.658
Total Switches	0.694 (0.653-0.736)	< 0.001	0.758 (0.708-0.811)	<0.001
Weeks Since Genotype	0.993 (0.989-0.997)	0.001^*^	0.993 (0.988-0.998)	0.008^*^

*P ≤ 0.05.

cOR, crude Odds Ratio; aOR, Adjusted Odds Ratio; GSS, Genotypic Susceptibility Score; VL, Viral Load.

### Model performance comparison and clinical benefit of the optimal model

**Figure 2:**
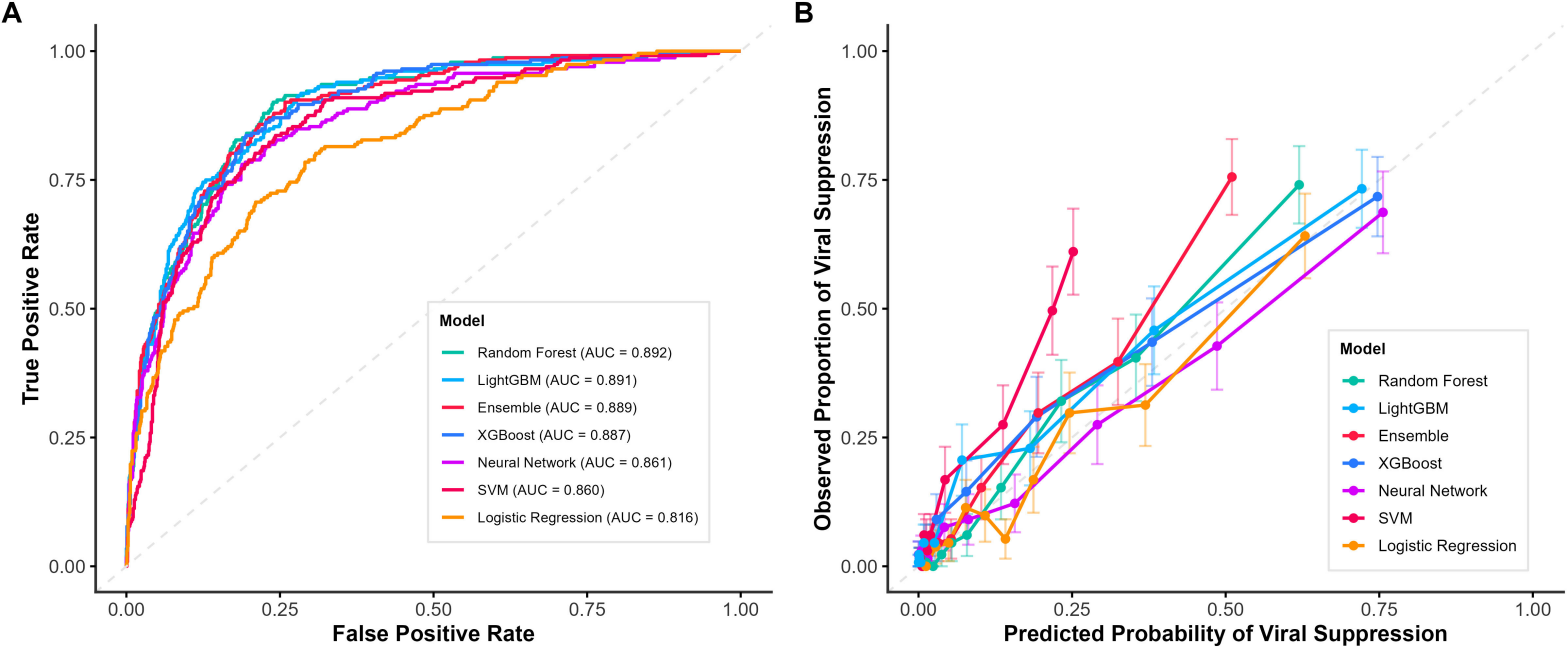
Performance evaluation of machine learning models for predicting viral suppression. **(A)** Receiver Operating Characteristic curves for the seven evaluated models on the testing set. **(B)** Calibration plots comparing predicted probabilities against observed fractions of viral suppression. The dashed diagonal gray line represents perfect calibration.

VIF results indicated no multicollinearity among the variables (**Supplementary Table 2**). Among the evaluated models, tree-based algorithms and the ensemble approach demonstrated superior discrimination (AUC range: 0.887–0.892) compared to the baseline Logistic Regression (AUC: 0.816) (**Figure 2A**). Ultimately, XGBoost was selected as the optimal model because it achieved a robust balance of accuracy (81.08%) and specificity (80.53%), coupled with excellent probability calibration (Brier score: 0.096; Calibration slope: 0.717), effectively avoiding the severe overconfidence observed in models like Random Forest (Slope = 1.210) (**Figure 2B**, **Supplementary Tables S3**, **S4**). Compared to the baseline model, the XGBoost model demonstrated substantial net clinical benefit (NRI: 0.490; IDI: 0.417). Reclassification Matrix analysis revealed the primary source of this benefit: XGBoost successfully corrected 61.90% (671/1,084) of actual treatment failures that were erroneously misclassified as “likely to succeed” by the baseline model.

### Key predictors and feature interactions

**Figure 3:**
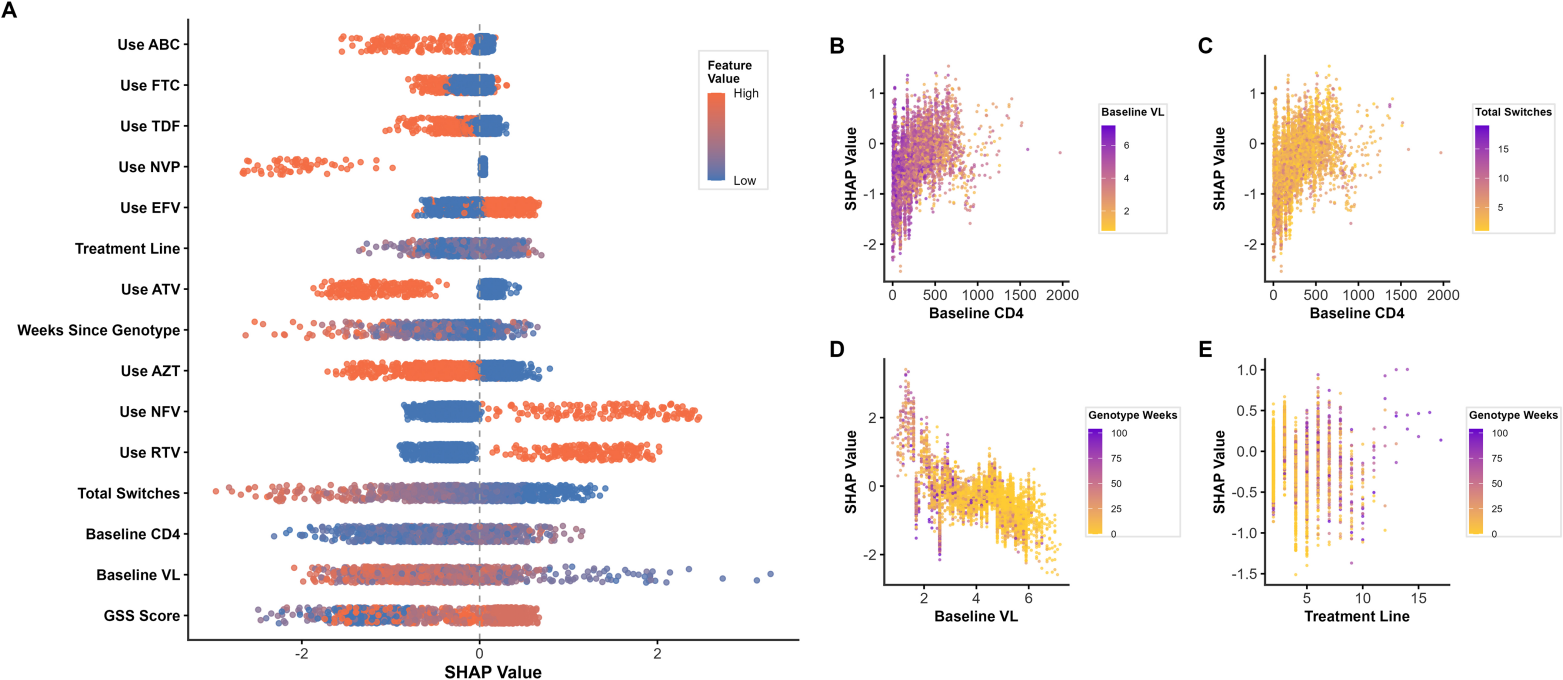
SHAP-based interpretability analysis of the optimal XGBoost model. **(A)** SHAP summary plot of the top 15 features ranked by global importance. Each dot represents a patient; color indicates the feature value (red = high, blue = low), and horizontal position indicates the SHAP value. **(B–E)** SHAP dependence plots illustrating interaction effects between key features: **(B)** Baseline CD4 count and Baseline Viral Load; **(C)** Baseline CD4 count and Total Switches; **(D)** Baseline Viral Load and Weeks Since Genotype; and **(E)** Treatment Line and Weeks Since Genotype. The color scale represents the value of the interacting feature.

The SHAP summary plot (**Figure 3A**) identified GSS score, total number of regimen switches, use of RTV, baseline VL, use of NFV, and baseline CD4 count as the top six predictors (**Supplementary Table 5**), demonstrating high consistency across validation folds (Mean Spearman’s rho = 0.9452; **Supplementary Table 6**). SHAP interaction analysis (**Figures 3B–E**) quantified the non-linear synergistic effects among these features. Notably, robust baseline immune function (CD4 > 233 cells/µL) significantly modulated and attenuated the negative impact of viral load: in this high-CD4 group, the predictive penalty for having a high viral load (> 4.4 log10 copies/mL) was minimal (difference in SHAP values [ΔSHAP] = 0.0195). Similarly, the negative impact of a heavy treatment history (> 3 prior switches) was largely attenuated in patients with robust immunity (ΔSHAP = 0.0072). Furthermore, a strong temporal decay was observed regarding genotypic testing: for heavily treated patients (> 3 lines), a recent genotypic result (≤ 1 week) strongly predicted treatment success (mean SHAP: -0.4560), whereas this predictive value dropped substantially if the test was older than 1 week (mean SHAP: -0.1495; absolute reduction = 0.3065). Finally, the SHAP dependence plot (**Supplementary Figure 2**) revealed a threshold effect for GSS, where scores ≥ 3 strongly drove positive predictions, while scores < 2 resulted in a sharp drop in predictive contribution.

### Sensitivity analysis of the optimal model

To evaluate the robustness of the model, this study constructed XGBoost models using three different outcome definitions, with thresholds of 50 copies/mL, 200 copies/mL, and 1000 copies/mL for virologic suppression, respectively (**Supplementary Figure 3**). The results showed AUCs of 0.887, 0.767, and 0.736, respectively. The 5-fold cross-validation results demonstrated highly consistent performance across the five validation folds, verifying that the model did not overfit and was robust to data perturbations (**Supplementary Figure 4**).

### Fairness assessment of the optimal model

Given the heterogeneity of clinical scenarios, this study evaluated the algorithmic fairness of the optimal model across different baseline VL and CD4^+^ T cell count subgroups (**Supplementary Figure 5**). The model demonstrated highly consistent predictive performance in both VL and immune status populations. For VL, the AUC was 0.914 (95% CI: 0.889-0.939) in the low group (VL ≤ 4.4 log10 copies/mL) and 0.864 (95% CI: 0.827-0.901) in the high group, with stable calibration (Brier Score: 0.092 vs. 0.099). For immune status, the AUCs were 0.871 (95% CI: 0.831-0911) in the severely immunocompromised group (CD4 < 200 cells/µL) and 0.894 (95% CI: 0.867-0.920) in the group with better immune function, showing no statistically significant difference.

### Individual prediction uncertainty assessment

This study mapped the association between predicted success rates and ESS (**Supplementary Figure 6**) through parameterized Bootstrap analysis. The results demonstrated a distinct, monotonic gradient in prediction confidence: individual certainty increased progressively with the predicted probability of success. In regions with extremely high predicted probabilities (>90%), the ESS reached its peak at a median of 16.9. Conversely, the median ESS was exceptionally low (3.1) in the highest-risk failure region (<10%) and gradually increased across the intermediate risk tiers (median ESS of 6.3 in the 40%–60% range). Across the entire cohort, the overall median ESS was 3.9. Notably, appropriately incorporating class-imbalance weights significantly expanded the uncertainty landscape for majority-class predictions, resulting in 60.8% of patients having an ESS below 5, with the lower quartile (25%) falling below.

### Feature ablation study on the superiority of the optimal model

The results of the feature ablation study (**Supplementary Figure 7**) demonstrated limitations in the predictive performance of models relying solely on immunological features or solely on treatment history features, with AUCs of 0.545 and 0.713, respectively. The model utilizing only virological features achieved higher discrimination (AUC = 0.875) but remained significantly lower than the integrated model (DeLong test, P = 0.037).

### Clinical utility and translational application of the optimal model

**Figure 4:**
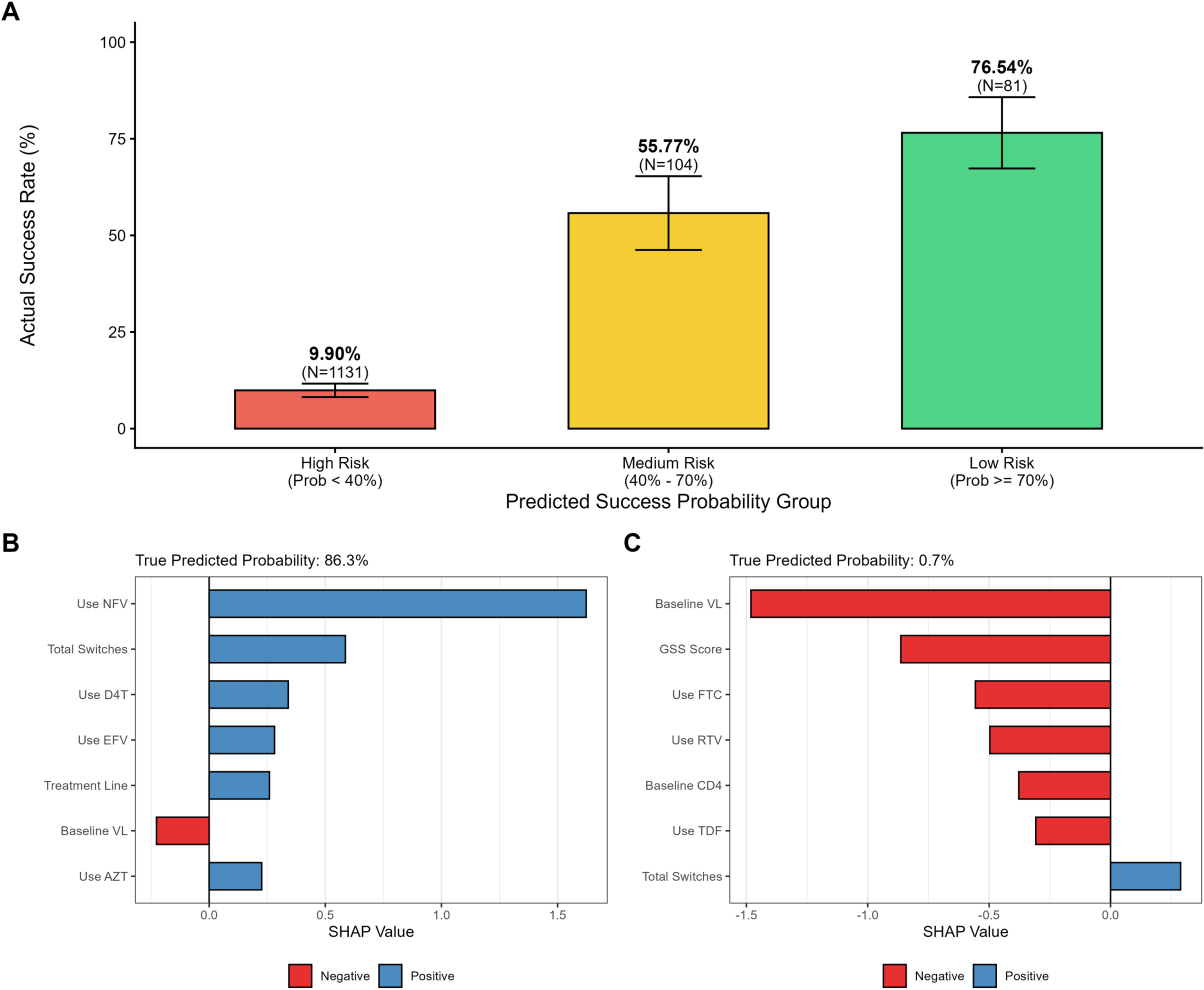
Risk stratification system and individual prediction examples using the XGBoost model. **(A)** Distribution of observed viral suppression rates across model-predicted risk strata (Low, Medium, and High). **(B)** SHAP force plot for a representative patient with a high predicted probability of treatment success. **(C)** SHAP force plot for a representative patient with a low predicted probability of treatment success.

The risk stratification system based on the optimal XGBoost model (**Figure 4**) demonstrated that the actual success rate in the low-risk group (predicted success probability ≥ 70%) was as high as 76.54%. In a clinical setting, this threshold can serve as a high-confidence reference to support physicians in confirming regimen viability. The intermediate-risk group (predicted probability 40%-70%) exhibited a 55.77% success rate, acting as a signal for clinicians to manually weigh patient-specific factors such as historical adherence. Conversely, the high-risk group (< 40%) yielded an actual success rate of only 9.90%, providing a critical early warning that encourages the clinical team to reassess the proposed combination or explore novel-mechanism drugs. DCA (**Supplementary Figure 8**) results indicated that within the 0-80% threshold range, the net benefit of using this XGBoost model to guide decision-making consistently outperformed default strategies. To facilitate practical deployment without replacing physician judgment, this study combined the optimal model with ESS to construct an assistive point-of-care calculator (**Supplementary Material Supplementary Figure 9**). This prototype demonstrates how the algorithm could be integrated into hospital Electronic Medical Records as a clinical decision support system, supplying clinicians with real-time risk assessments to augment, rather than replace, their final therapeutic decisions.

## Discussion

This study used standardized, de-identified data from 18 clinical trials to construct a prediction model for salvage therapy outcomes in patients with virologic failure, in accordance with the TRIPOD+AI statement. The results indicated that the XGBoost-based prediction model achieved significant improvements in discrimination, specificity, and calibration compared to the traditional GSS logistic regression model. This superior performance suggests that precise salvage therapy prediction cannot rely solely on isolated drug activity calculations. By capturing the complex, multidimensional, nonlinear interactions among viral genotypic resistance, host immune status, and baseline disease burden, the XGBoost framework offers a comprehensive clinical perspective that effectively minimizes the risks of ineffective regimen switching.

To ensure robust benchmarking beyond the traditional GSS system, we compared our XGBoost framework against both internally developed contemporary ML algorithms and externally published models. Our internal comprehensive evaluations demonstrated the superiority of XGBoost over contemporary algorithms such as Random Forest, LightGBM, and Neural Networks, particularly in its ability to balance discrimination with probability calibration. Furthermore, external benchmarking highlights its clinical validity. Early research using the European EuResist dataset achieved comparable AUC ranges via classifier fusion of genotype and clinical data (13); however, our framework advances this paradigm by providing patient-level SHAP interpretability and strictly adhering to TRIPOD+AI standards. Furthermore, while Revell et al. demonstrated the utility of ML without genotypic data in resource-limited settings (14), and Yehadji et al. recently achieved a maximum AUC of 0.82 in a West African cohort using contemporary ML methods (15), our XGBoost model demonstrates superior discrimination with an AUC of 0.887. This performance is particularly robust given that we utilized the strictest virologic suppression threshold (<50 copies/mL), directly addressed class imbalance via algorithm-level weighting rather than synthetic oversampling, and empirically verified the necessity of integrating virological, immunological, and treatment history features.

Using SHAP interaction analysis, this study quantified the nonlinear interaction between PLWHs’ immune status and viral dynamics. The results showed that baseline CD4+ T cell counts modify the predictive weight of baseline viral load on salvage therapy outcomes. In PLWH with better immune function, a high viral load only weakly predicts treatment failure (SHAP decrease of only 0.0195). Clinically, this suggests that a robust immune system partially modulates and stabilizes the negative impact of high viral replication. This observation aligns with dynamic modeling studies showing robust immune reconstitution supports durable virologic suppression (25). Conversely, in PLWH with severely compromised immunity, this immune modulatory effect diminishes. Elevated baseline viral loads then translate directly into a high risk of failure. These findings emphasize the need for a stratified intervention strategy. PLWH with lower CD4 counts need optimized drug regimens and closer clinical monitoring, as they lack the physiological margin for error.

In addition to virus-host interactions, the model also identified temporal decay in genotypic data. As resistance test results age, their predictive weight in the model decreases. Biologically, during periods without effective drug pressure or with poor adherence, plasma HIV quasispecies may revert to wild type or undergo genetic drift (26, 27). Outdated genotypic test results then lose predictive relevance and may not reflect the resistance profile of the current dominant strain in heavily treated PLWH. This algorithmic finding supports recent large-cohort studies showing that the detection rate of resistance mutations declines with prolonged drug discontinuation. These results reinforce the clinical need to obtain immediate genotypic resistance testing before starting salvage therapy (2).

In addition to physiological indicators, this study revealed the impact of treatment history complexity on salvage therapy outcomes. GLMM analysis showed that the total number of regimen switches was a strong negative predictor. Mechanistically, this highlights a blind spot of the traditional GSS scoring system: routine genotypic testing is based solely on plasma RNA and captures genetic information only from the replicating dominant strain, without detecting proviral DNA integrated into the host cell genome (28). A history of frequent switching implies a complex history of cumulative resistance mutations. Although these archived mutations are not detected by current RNA sequencing, they may be reactivated from the reservoir and lead to treatment failure when drug selection pressure changes (27). Recent studies confirm that, in patients experiencing multiple treatment failures, the detection rate of resistance mutations in proviral DNA is significantly higher than in plasma RNA (29), and this discrepancy increases with treatment duration (30). Review studies emphasize that for PLWH with viral suppression or low-level viremia, DNA genotype testing is essential to assess the resistance reservoir (31). Therefore, identifying the total number of switches as a key feature in our model serves as an algorithmic proxy for cumulative resistance burden.

Clinically, this concept of cumulative resistance burden provides strong evidence for reconstructing medication strategies for PLWH with multiple switches. Traditional treatment paradigms often reserve drugs with novel mechanisms as a last resort. However, consistent with previous research (32, 33), our findings suggest that as the number of switches increases, the probability of treatment success with traditional ART regimens drops sharply. Recent clinical trial data support an alternative approach: the 104-week CAPELLA study showed that the capsid inhibitor Lenacapavir achieved an 83% viral suppression rate in multidrug-resistant PLWH (34), and 240-week data on Fostemsavir similarly confirmed long-term survival benefits in heavily treatment-experienced PLWH (35, 36). This indicates that when facing PLWH with complex switching histories, clinicians should proactively advance novel-mechanism drugs to break the vicious cycle of incomplete viral suppression and mutation accumulation, rather than continuing to trial traditional classes that are likely to share cross-resistance.

To evaluate whether the model can correct erroneous clinical decisions, this study calculated the net clinical benefit using reclassification analysis. By addressing the aforementioned biological blind spots of isolated GSS scoring, XGBoost successfully reclassified 61.90% (671/1,084) of actual treatment failures that were erroneously judged “likely to succeed” by the baseline model. This pronounced improvement in specificity endows the model with a crucial decision-support safeguard function. In multidrug-resistant PLWH management, the greatest danger is the continued use of ineffective ART regimens leading to functional monotherapy. By accurately identifying these high-risk failures, the model empowers clinicians to block the initiation of ineffective treatments (14), thereby preventing unnecessary drug toxicities, protecting drug-sensitive viral sites, and halting the continuous accumulation of cross-resistance (10).

Beyond individual patient reclassification, the model demonstrated the robust statistical reliability required for high-stakes decision-making. Decision Curve Analysis (DCA) confirmed that within the 0%-80% probability range, the net benefit of using the XGBoost model consistently outperformed default “treat-all” or “treat-none” strategies. Recent comments on the TRIPOD+AI statement emphasize that, for AI tools guiding clinical decisions, the accuracy of predicted probabilities is the cornerstone of building trust (20, 37). The excellent calibration performance of the XGBoost model constructed here indicates that its predicted probabilities closely align with actual incidence rates, effectively overcoming the tendency toward overconfidence common in complex machine learning models such as Random Forest (38). This means that when the model provides a predicted success probability, clinicians can be confident it reflects real clinical risk rather than an overconfident artifact caused by poor model calibration.

Addressing the issue of algorithmic bias, which is of increasing concern in the modern medical AI field, this study confirmed through subgroup analysis that the AUC and calibration of the optimal model remained highly stable across different viral load groups and degrees of immune compromise, without the systematic bias in severe marginalized groups noted in previous studies (16). This stable algorithmic fairness is largely attributed to this study setting the predicted treatment success viral load threshold at the strictest standard of 50 copies/mL. Previous studies have shown that outcomes between 50 and 1000 copies/mL are confounded by significant non-biological noise, such as adherence fluctuations and transient viral blips, whereas achieving “complete viral load suppression” is a biological signal with a high signal-to-noise ratio driven by drug efficacy (39). The sensitivity analysis results showed that using 50 copies/mL as the threshold for treatment success yielded significantly better performance than 200 copies/mL and 1000 copies/mL, and also revealed the noisy nature of the 50–1000 copies/mL range. Large cohort studies indicate that even if post-treatment VL is maintained at 50–200 copies/mL, the risk of subsequent virologic failure in PLWH is still 2.5 times higher than that of viral suppressors (40), and persistent LLV has been confirmed to be closely related to chronic inflammation and severe non-AIDS-defining complications (41). In the context where “U=U” has become a global consensus for HIV prevention and treatment (8, 9), the results of this study suggest that resistance prediction models should not use lenient viral load thresholds for the sake of achieving high AUC. Using 50 copies/mL as the threshold for treatment success not only ensures the fairness of the model algorithm across patients with different disease severities but also reduces the continuous accumulation of the resistance reservoir (42) and lowers the risk of long-term complications.

To translate algorithms into clinical practice, this study developed a visual platform to predict salvage therapy success rates and introduced ESS analysis to quantify the uncertainty of individual predictions. The optimal model constructed in this study demonstrated a distinct gradient in ESS. While confidence was highest for predicting success, the ESS was exceptionally low in the extreme high-risk failure region and remained moderately low in the decision gray zone due to data sparsity and complex patient heterogeneity (43). This finding not only outlines the trust boundary for AI-assisted decision-making but also provides quantitative operational guidelines for a new “Human-in-the-loop” diagnosis-and-treatment mode. Traditional views often worry that the emergence of AI will replace doctors, but the results of this study suggest that AI’s core value lies in precisely defining the algorithm’s Scope of Ignorance. That is, when the model indicates a low ESS or is in the decision gray zone, it is actually signaling a need for clinician takeover, suggesting that manual adjustment, combining external factors such as patient adherence, drug interactions, and complications, is needed at this time. Furthermore, the risk stratification system constructed using the optimal model showed that the actual success rate in the high-risk group was only 9.90%. For such PLWH, merely adjusting existing classes of drugs may yield little effect. This demonstrates the intervention limit based on existing data models, suggesting that the value of AI is no longer to recommend drugs for this high-risk population, but to support clinicians in deciding to switch tracks to introduce drugs with entirely new mechanisms that have lower background resistance values (44, 45) or higher clinical benefits (34–36). Through this stratification strategy and uncertainty assessment, the model developed in this study is not only a predictive tool but also a navigator that can guide resource-allocation optimization, contributing to the implementation and advancement of evidence-based precision medicine in the HIV field (46).

It must be emphasized that SHAP analysis reflects statistical associations based on data distributions, not direct biological causality. For example, the high predictive importance of RTV in our model reflects its clinical role as a CYP3A4 pharmacokinetic enhancer for other protease inhibitors (47, 48), rather than an independent antiviral mechanism. Consequently, its high SHAP weight serves as a clinical proxy for complex, boosted PI-based salvage regimens. To prevent over-extrapolation, the interpretation of observed algorithmic interactions, such as the immune modulatory effect, was comprehensively validated using established pathophysiological evidence rather than relying solely on algorithmic outputs.

Although this study developed a robust prediction model, several limitations exist. First, the strict inclusion criteria of clinical trials (e.g., excluding severe psychiatric illnesses or complex comorbidities) may limit external generalizability. While this idealized dataset precisely captures pure virus-drug interactions, real-world clinical heterogeneity is higher. Second, strict database de-identification precluded fairness assessments based on demographics (sex, race, socioeconomic status), as recommended by TRIPOD+AI; however, we mitigated this by conducting subgroup “clinical fairness checks.” Third, lacking direct adherence data, the model reflects an “efficacy ceiling” assuming optimal adherence. Given the increasing use of high-forgiveness and long-acting drugs, predicting biological drug efficacy aligns with modern precision medicine needs, reducing the urgency for complex behavioral variables. Fourth, historical data limit coverage of novel mechanisms such as capsid inhibitors (Lenacapavir) or long-acting CAB/RPV. To address this, our model can serve as a “foundation model,” allowing rapid adaptation to new therapies via transfer learning with minimal new clinical data. Fifth, missing accurate HIV-1 subtype data may confound the inherent differences in genetic resistance barriers; future studies should validate cross-subtype generalizability.

## Conclusion

This study successfully developed and validated an XGBoost prediction model strictly adhering to TRIPOD+AI standards. By integrating multidimensional features and utilizing SHAP interaction analysis, the model overcomes the linear limitations of traditional genotypic susceptibility scores, precisely quantifying the non-linear immune modulatory effect of host immune status and the negative impact of treatment history complexity on viral suppression. The results confirm that this model demonstrates excellent discrimination, calibration, and net clinical benefit in predicting complete virologic suppression during HIV-1 salvage therapy. It effectively identifies and corrects traditional misjudgments regarding ineffective regimens, acting as a clinical safeguard to prevent further resistance accumulation. Furthermore, the robust algorithmic fairness demonstrated in highly vulnerable subgroups, combined with the quantitative assessment of individual prediction uncertainty, provides clinicians with a trustworthy, transparent, and precise auxiliary tool. This supports complex decision-making in multidrug-resistant HIV management, facilitating a critical paradigm shift from empirical trial-and-error approaches to stratified precision interventions.

## Data Availability

Publicly available datasets were analyzed in this study. Data was derived from the publicly available Stanford HIVDB. The data and analysis code is available from the link: https://doi.org/10.6084/m9.figshare.31921962.
