# Supplementary material for: Machine learning quantifies immuno-virological interactions: a TRIPOD+AI compliant prediction model for HIV-1 salvage therapy outcomes
Source: Front Immunol. 2026 Aug 5;17:1846559. doi: 10.3389/fimmu.2026.1846559 (PMC13485717; doi:10.3389/fimmu.2026.1846559)
Supplement: Supplementary file 1 [file SupplementaryFile1.docx]

**Supplemental Materials 1**

**Contents**

[**Figure S1 Feature Correlation Matrix	2**](#_Toc233749173)

[**Figure S2 SHAP Dependence Plot for Genotypic Susceptibility Score	3**](#_Toc233749174)

[**Figure S3 Sensitivity Analysis ROC Curves	4**](#_Toc233749175)

[**Figure S4 Cross-Validation ROC Curves	5**](#_Toc233749176)

[**Figure S5 Algorithmic Fairness Across Subgroups	6**](#_Toc233749177)

[**Figure S6 Distribution of Effective Sample Size (ESS)	7**](#_Toc233749178)

[**Figure S7 Ablation Study of Feature Domains	8**](#_Toc233749179)

[**Figure S8 Decision Curve Analysis (DCA)	9**](#_Toc233749180)

[**Figure S9 Online Risk Calculator Interface	10**](#_Toc233749181)

[**Table S1 Model Hyperparameters	11**](#_Toc233749182)

[**Table S2 Multicollinearity Diagnostics	12**](#_Toc233749183)

[**Table S3 Hosmer-Lemeshow Calibration Test	13**](#_Toc233749184)

[**Table S4 Model Performance Metrics	14**](#_Toc233749185)

[**Table S5 SHAP Feature Importance Ranking	16**](#_Toc233749186)

[**Table S6 SHAP Feature Importance Stability	17**](#_Toc233749187)

# Figure S1 Feature Correlation Matrix

Heatmap displays Pearson correlation coefficients between key predictor variables included in the model. Red indicates positive correlation, Blue indicates negative correlation, and White indicates no correlation.

# Figure S2 SHAP Dependence Plot for Genotypic Susceptibility Score

The x-axis represents the GSS score derived from the Stanford HIVDB algorithm, and the y-axis represents the SHAP value (log-odds impact on prediction). Each dot corresponds to an individual patient. The color gradient indicates the Baseline Viral Load (Blue: Low, Red: High).

# Figure S3 Sensitivity Analysis ROC Curves

Comparison of the XGBoost model's predictive performance across three viral load thresholds for treatment success.

# Figure S4 Cross-Validation ROC Curves

ROC curves derived from trial-balanced 5-fold cross-validation. Stratification by Clinical Trial ID ensures representative distributions across all 18 diverse trials.

# Figure S5 Algorithmic Fairness Across Subgroups

Forest plot illustrates XGBoost predictive performance stratified by key clinical characteristics.

# Figure S6 Distribution of Effective Sample Size (ESS)

# Figure S7 Ablation Study of Feature Domains

Predictive performance comparison between the Integrated/Optimal Model (red line) and three single-domain partial models (virological, immunological, and treatment history).

# Figure S8 Decision Curve Analysis (DCA)

The plot compares the Net Benefit (y-axis) of different treatment strategies across a range of Threshold Probabilities (x-axis). The Blue line represents the XGBoost model. The Grey line represents the "Treat All" strategy, and the Green line represents "Treat None".

# Figure S9 Online Risk Calculator Interface

# Table S1 Model Hyperparameters

| **Model** | **Parameter Configuration** | **Value Description** |
| --- | --- | --- |
| Logistic Regression | Family | Binomial (Logistic) |
|  | Class Weighting | Observation weights applied (Minority: 3.91, Majority: 1) |
| Random Forest | Number of Trees (num.trees) | 500 |
|  | Probability Mode | TRUE |
|  | Class Weighting (case.weights) | Observation weights applied (Minority: 3.91, Majority: 1) |
| XGBoost | Booster | gbtree |
|  | Objective | binary:logistic |
|  | Grid Search Parameters | max_depth: c(4, 6), eta: c(0.05, 0.1) |
|  | Static Tree Parameters | min_child_weight: 3, subsample: 0.8, colsample_bytree: 0.8 |
|  | Early Stopping & CV | 5-fold CV, early_stopping_rounds: 50 |
|  | Number of Rounds (nrounds) | Optimized via CV (max 300) |
|  | Evaluation Metric | AUC |
|  | Class Weighting (scale_pos_weight) | 3.91 (Algorithm-level compensation) |
| LightGBM | Objective | binary |
|  | Number of Leaves (num_leaves) | 31 |
|  | Learning Rate | 0.05 |
|  | Number of Rounds | 500 |
|  | Force Col-wise | TRUE |
|  | Class Weighting (scale_pos_weight) | 3.91 (Algorithm-level compensation) |
| Support Vector Machine | Kernel | Radial Basis Function |
|  | Probability Mode | TRUE |
|  | Class Weighting (class.weights) | Majority: 1, Minority: 3.91 |
| Neural Network | Architecture | Single hidden layer feed-forward network (nnet) |
|  | Hidden Layer Neurons (size) | 10 |
|  | Regularization | Weight decay (L2 regularization): 0.05 |
|  | Max Iterations (maxit) | 200 |
|  | Output Activation | Logistic (linout = FALSE) |
|  | Class Weighting | Observation weights applied (Minority: 3.91, Majority: 1) |
| Ensemble | Fusion Strategy | Soft Voting (Arithmetic mean of predicted probabilities from Random Forest, XGBoost, SVM, and Neural Network) |

# Table S2 Multicollinearity Diagnostics

| **Feature** | **VIF** | **Feature** | **VIF** |
| --- | --- | --- | --- |
| Use FTC | 8.147521025 | Use Unknown Regimen | 2.643456106 |
| Use 3TC | 6.430902935 | Use NFV | 2.264680220 |
| Use TDF | 5.946608914 | Use ATV | 2.106489918 |
| Use AZT | 5.275372654 | Total Switches | 2.104594965 |
| GSS Score | 4.441728878 | Weeks Since Genotype | 1.971168482 |
| Use DDI | 3.922107846 | Baseline VL | 1.773103110 |
| Use D4T | 3.903159219 | Use ABC | 1.685718175 |
| Use RTV | 3.333738343 | Recycled 3TC/FTC | 1.636456599 |
| Use EFV | 3.134836606 | Use IDV | 1.600980737 |
| Treatment Line | 3.040086606 | Baseline CD4 | 1.263653326 |
| Use LPV | 2.729549379 | Use NVP | 1.000000422 |

**VIF:** Variance Inflation Factor; **FTC:** Emtricitabine; **3TC:** Lamivudine; **TDF:** Tenofovir disoproxil fumarate; **AZT:** Zidovudine; **GSS:** Genotypic Susceptibility Score; **DDI:** Didanosine; **D4T:** Stavudine; **RTV:** Ritonavir; **EFV:** Efavirenz; **LPV:** Lopinavir; **NFV:** Nelfinavir; **ATV:** Atazanavir; **VL:** Viral Load; **ABC:** Abacavir; **IDV:** Indinavir; **CD4:** CD4+ T cell count; **NVP:** Nevirapine;

# Table S3 Hosmer-Lemeshow Calibration Test

| **Model** | **χ2** | **P Value** |
| --- | --- | --- |
| Logistic Regression | 360.23 | <0.001 |
| Random Forest | 186.91 | <0.001 |
| XGBoost | 140.28 | <0.001 |
| LightGBM | 146.06 | <0.001 |
| Support Vector Machine | 15.10 | 0.0572 |
| Neural Network | 493.65 | <0.001 |
| Ensemble | 100.60 | <0.001 |

**Note:** The Hosmer-Lemeshow (H-L) test yielded significant P-values (< 0.001), a recognized limitation in large datasets (n > 5000) where the test becomes overly sensitive to negligible deviations. Furthermore, probability recalibration for class-imbalance alters decile distributions, inherently inflating test statistics.

P ≤ 0.05 was considered statistically significant.

# Table S4 Model Performance Metrics

| **Model** | **AUC** | **Accuracy** | **Sensitivity** | **Specificity** | **PPV** | **NPV** | **F1** | **Kappa** | **Brier Score** | **Calibration Intercept** | **Calibration Slope** |
| --- | --- | --- | --- | --- | --- | --- | --- | --- | --- | --- | --- |
| Random Forest | 0.8923 (0.8706-0.9141) | 0.7857 (0.7625-0.8076) | 0.9009 (0.8550-0.9361) | 0.7611 (0.7345-0.7862) | 0.4466 (0.4009-0.4929) | 0.9729 (0.9596-0.9827) | 0.5971 | 0.4729 | 0.0940 | 0.471 | 1.210 |
| LightGBM | 0.8907 (0.8682-0.9132) | 0.7546 (0.7304-0.7776) | 0.9138 (0.8700-0.9465) | 0.7205 (0.6927-0.7470) | 0.4117 (0.3688-0.4555) | 0.9750 (0.9617-0.9847) | 0.5676 | 0.4287 | 0.0937 | 0.158 | 0.703 |
| Ensemble | 0.8890 (0.8666-0.9114) | 0.7698 (0.7460-0.7923) | 0.9009 (0.8550-0.9361) | 0.7417 (0.7145-0.7675) | 0.4274 (0.3831-0.4726) | 0.9722 (0.9586-0.9823) | 0.5798 | 0.4477 | 0.0991 | 0.745 | 1.139 |
| XGBoost | 0.8873 (0.8650-0.9096) | 0.8108 (0.7886-0.8316) | 0.8362 (0.7822-0.8814) | 0.8054 (0.7805-0.8285) | 0.4790 (0.4294-0.5289) | 0.9583 (0.9432-0.9703) | 0.6091 | 0.4962 | 0.0955 | 0.101 | 0.717 |
| Neural Network | 0.8610 (0.8341-0.8878) | 0.8062 (0.7838-0.8273) | 0.7802 (0.7213-0.8317) | 0.8118 (0.7872-0.8347) | 0.4701 (0.4194-0.5213) | 0.9452 (0.9286-0.9589) | 0.5867 | 0.4701 | 0.0999 | -0.316 | 0.686 |
| Support Vector Machine | 0.8597 (0.8336-0.8857) | 0.7713 (0.7476-0.7937) | 0.8362 (0.7822-0.8814) | 0.7574 (0.7307-0.7826) | 0.4245 (0.3787-0.4713) | 0.9558 (0.9398-0.9685) | 0.5631 | 0.4298 | 0.1274 | 1.289 | 1.003 |
| Logistic Regression | 0.8160 (0.7858-0.8461) | 0.7751 (0.7515-0.7974) | 0.7069 (0.6438-0.7646) | 0.7897 (0.7642-0.8136) | 0.4184 (0.3690-0.4689) | 0.9264 (0.9076-0.9424) | 0.5256 | 0.3907 | 0.1104 | -0.032 | 1.033 |

**AUC:** Area Under the Receiver Operating Characteristic Curve

**PPV:** Positive Predictive Value

**NPV:** Negative Predictive Value

**F1:** F1-Score, the harmonic mean of sensitivity and positive predictive value

**Kappa:** Cohen’s Kappa Coefficient

# Table S5 SHAP Feature Importance Ranking

| **Rank** | **Feature** | **SHAP Value*** | **Rank** | **Feature** | **SHAP Value*** |
| --- | --- | --- | --- | --- | --- |
| 1 | GSS Score | 0.646169950 | 12 | Use NVP | 0.132833846 |
| 2 | Baseline VL | 0.598850192 | 13 | Use TDF | 0.130483002 |
| 3 | Baseline CD4 | 0.528968245 | 14 | Use FTC | 0.118001569 |
| 4 | Total Switches | 0.515112853 | 15 | Use ABC | 0.115971998 |
| 5 | Use RTV | 0.501386424 | 16 | Use D4T | 0.095720873 |
| 6 | Use NFV | 0.450627329 | 17 | Use DDI | 0.093052150 |
| 7 | Use AZT | 0.365134541 | 18 | Use LPV | 0.082076383 |
| 8 | Weeks Since Genotype | 0.337316217 | 19 | Use 3TC | 0.079729698 |
| 9 | Use ATV | 0.276102382 | 20 | Use IDV | 0.070548539 |
| 10 | Treatment Line | 0.248846761 | 21 | Recycled 3TC FTC | 0.057901430 |
| 11 | Use EFV | 0.223168750 | 22 | Use Unknown Regimen | 0.046757116 |

***:** Mean Absolute SHAP Value

**SHAP:** SHapley Additive exPlanations; **GSS:** Genotypic Susceptibility Score; **VL:** Viral Load; **CD4:** CD4+ T cell count; **RTV:** Ritonavir; **NFV:** Nelfinavir; **AZT:** Zidovudine; **ATV:** Atazanavir; **EFV:** Efavirenz; **NVP:** Nevirapine; **TDF:** Tenofovir disoproxil fumarate; **FTC:** Emtricitabine; **ABC:** Abacavir; **D4T:** Stavudine; **DDI:** Didanosine; **LPV:** Lopinavir; **3TC:** Lamivudine; **IDV:** Indinavir.

# Table S6 SHAP Feature Importance Stability

| **Features** | **Fold 1** | **Fold 2** | **Fold 3** | **Fold 4** | **Fold 5** |
| --- | --- | --- | --- | --- | --- |
| Baseline_VL | 1 | 2 | 1 | 2 | 2 |
| GSS_Score | 2 | 1 | 2 | 1 | 1 |
| Baseline_CD4 | 3 | 3 | 3 | 3 | 3 |
| Total_Switches | 4 | 5 | 6 | 5 | 4 |
| Use_RTV | 5 | 4 | 5 | 4 | 6 |
| Use_NFV | 6 | 6 | 4 | 6 | 5 |
| Weeks_Since_Genotype | 7 | 8 | 7 | 7 | 7 |
| Use_AZT | 8 | 7 | 8 | 8 | 8 |
| Use_ATV | 9 | 11 | 9 | 11 | 10 |
| Treatment_Line | 10 | 10 | 10 | 9 | 11 |

**Note:** Top 10 features and their importance rankings across 5-fold cross-validation. The high consistency (Mean Spearman's rho = 0.9452) indicates the robustness of the model's interpretability.

**SHAP:** SHapley Additive exPlanations; **VL:** Viral Load; **GSS:** Genotypic Susceptibility Score; **CD4:** CD4+ T cell count; **RTV:** Ritonavir; **NFV:** Nelfinavir; **AZT:** Zidovudine; **ATV:** Atazanavir.
